# Intracavitary course of right coronary artery

**DOI:** 10.1007/s12471-019-1264-z

**Published:** 2019-04-08

**Authors:** L. J. Bouhuijzen, J. J. Kardux, R. L. Braam

**Affiliations:** 1 Department of Cardiology, Thoraxcentrum Twente, Medisch Spectrum Twente, Enschede, The Netherlands; 2 Department of Radiology, Gelre Ziekenhuizen, Apeldoorn, The Netherlands; 3 Department of Cardiology, Gelre Ziekenhuizen, Apeldoorn, The Netherlands

**Fig. 1a–c:**
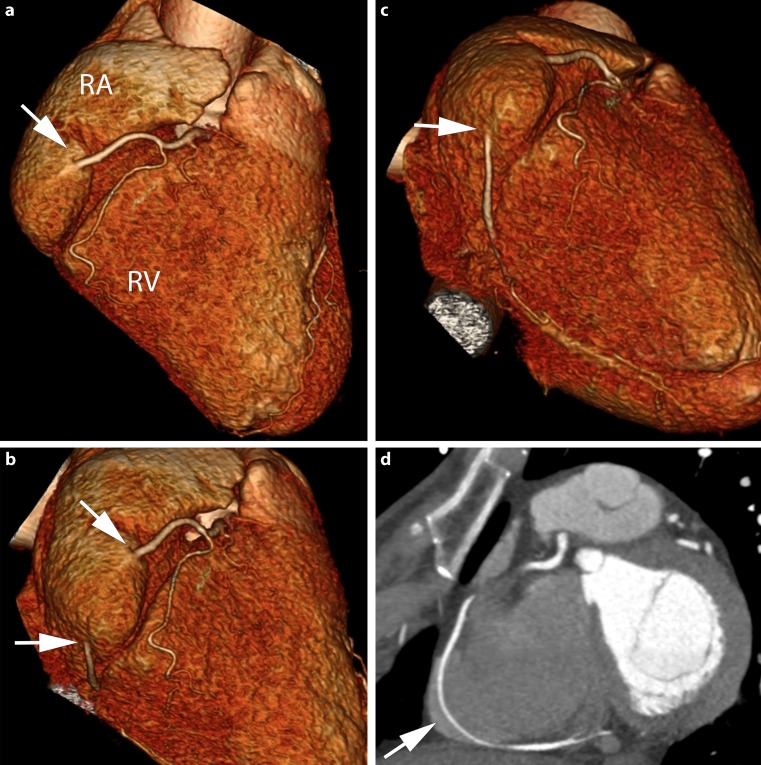
Intra-atrial course of the right coronary artery (RCA) using volume-rendered 3D reconstructions. **a** *Arrow* showing the RCA entering the right atrium (*RA*); the right ventricle (*RV*) is shown as well. **b**, **c** *Arrows* showing the RCA entering and exiting the RA. **d** Coronal view of the RCA, the *arrow* indicates the intra-atrial course

A 64-year-old woman was referred to the cardiology department for evaluation of atypical chest pain. A cardiac computed tomography (CT) scan demonstrated a calcium score of zero and no coronary stenosis. As an incidental finding, the scan revealed an anomalous course of a 4-cm segment of the right coronary artery (RCA) within the right atrium. The CT scan is shown in Fig. 1. No abnormalities were noted in the other coronary arteries and branches.

An intra-atrial or intracavitary course of the RCA is rare; the incidence is estimated at 0.09–0.1% [1]. Although this anomaly does not account for the patient’s symptoms, it is of significance for various potential interventions. RCA injury may occur during pacemaker implantation, right heart catheterisation, electrophysiological studies and ablations [2]. Disruption can lead to myocardial ischaemia and left-to-right shunting. Furthermore, difficulties may arise during coronary artery bypass surgery with regard to vessel localisation or bypass grafting.
